# The Normalized Reduced Form and Cell Mathematical Tools for Lattice Analysis—Symmetry and Similarity

**DOI:** 10.6028/jres.108.039

**Published:** 2003-12-01

**Authors:** Alan D. Mighell

**Affiliations:** National Institute of Standards and Technology, Gaithersburg, MD 20899-8520

**Keywords:** identification, lattice-matching strategies, lattice relationships, lattice similarity, metric lattice, normalized reduced cell and form, symmetry

## Abstract

To intelligently and effectively use crystallographic databases, mathematical and computer tools are required that can elucidate diverse types of intra- and interlattice relationships. Two such tools are the *normalized* reduced form and normalized reduced cell. Practical experience has revealed that the first tool—the normalized reduced form—is very helpful in establishing lattice metric symmetry as it enables one to readily deduce significant relationships between the elements of the reduced form. Likewise research with crystallographic databases has demonstrated that the second tool—the normalized reduced cell—plays a vital role in determining metrically *similar* lattices. Knowledge of similar lattices has practical value in solving structures, in assignment of structure types, in materials design, and in nano-technology. In addition to using the reduced cell, it is recommended that lattice-matching strategies based on the normalized reduced cell be routinely carried out in database searching, in data evaluation, and in experimental work.

## 1. Introduction

The various crystallographic databases [1] now available constitute a large, comprehensive, and rapidly growing scientific resource, serving as an invaluable source of data for the intelligent design of materials, for crystal engineering, and for nanotechnology. To evaluate data entering these databases and to intelligently and effectively use this resource, diverse mathematical tools are required that can establish intralattice relationships or elucidate various types of interlattice relationships.

Two such tools are the *normalized* reduced form and the *normalized* reduced cell—tools that are ideal for elucidating certain types of intra- and interlattice relationships. For example, with the normalized reduced form, one can determine lattice-metric symmetry and deduce other types of intralattice relationships. With the normalized reduced cell, one can determine metrically *similar* lattices^1^ via lattice matching techniques against the lattices in the crystallographic databases. Practical experience has revealed that these tools are very useful for routine and complex lattice analyses. Before proceeding with applications of these tools, it is necessary to define the normalized reduced cell and form.

### 1.1 Definitions

The reduced cell is a unique primitive cell of the lattice, which is based on the three shortest lattice translations. For the precise mathematical definition of the reduced cell and form and for procedures to calculate this cell, see [2] and NBS Technical Note 1290 [3].

The normalized reduced cell of a lattice is determined simply by dividing the cell edges of the reduced cell by the *a*-cell edge. The normalized reduced form is calculated from the normalized reduced cell and is defined by the vector dot products of the normalized reduced cell edge vectors:

$$\begin{array}{ccc} a\cdot a & b\cdot b & c\cdot c\\ b\cdot c & a\cdot c & a\cdot b \end{array}$$

As an example, consider the reduced cell for a typical triclinic crystal structure reported in the recent literature [4]:

$$\begin{array}{ccc} a_{t}=9.6907\text{Å} & b_{t}=10.3119\text{Å} & c_{t}=11.2549\text{Å}\\ \alpha_{t}=63.954{}^{\circ} & \beta_{t}=70.282{}^{\circ} & \gamma_{t}=87.414{}^{\circ} \end{array}$$

The corresponding normalized reduced cell and form are:

$$cell:\;\begin{array}{ccc} a=1.0000 & b=1.0641 & c=1.1614\\ \alpha =63.954{}^{\circ} & \beta =70.282{}^{\circ} & \gamma =87.414{}^{\circ} \end{array}$$

$$\begin{array}{cccc} Form: & \begin{array}{c} a\cdot a\\ b\cdot c \end{array} & \begin{array}{c} b\cdot b\\ a\cdot c \end{array} & \begin{array}{c} c\cdot c\\ a\cdot b \end{array} \end{array}=\begin{array}{ccc} 1.000 & 1.132 & 1.349\\ 0.543 & 0.392 & 0.048 \end{array}$$

The fact that there is no specialization^2^ in the normalized reduced form shows that the metric lattice is triclinic.

## 2. Discussion and Applications

The reduced form and cell have long been used in lattice metric symmetry determination and identification, respectively. Although the reduced form can be used in the symmetry checks discussed below, the normalized reduced form has the advantage in that it makes the interrelationships—and specialization—of the elements of the reduced form more transparent. Recognition of matrix-element specialization is a basis of symmetry determination as well as for investigations of many other lattice-related phenomena. Likewise, although Crystal Data Determinative Ratios [5] may be used to locate similar lattices within a given crystal system, the normalized reduced cell provides the logical basis for a far more powerful and comprehensive lattice-matching technique which is crystal system independent and conceptually parallel to techniques based on matching reduced cells. Details of the application of the normalized reduced form and cell for symmetry determination and for the determination lattice-metric similarity are outlined below.

### 2.1 Symmetry Determination via the Normalized Reduced Form (NRF)

The normalized reduced form (NRF) is a practical tool, which can be used—in conjunction with the matrix method [6]—for metric symmetry determination. With the NRF one can readily determine the metric symmetry of the lattice by matching it against a table of the 44 reduced forms [7].

To illustrate, Table 1 herein presents the 13 reduced forms corresponding to the centered monoclinic lattices. Typical examples of the NRFs are given that have been derived from cell constants published in recent issues of *Acta Crystallographica Section E*. Once normalized, the pattern of the relationships of dot products in the NRF is easy to ascertain. From the examples, one can see that it is especially easy to determine the reduced form number (first column) by matching a given NRF against the characteristic reduced form matrices presented in the second through fourth columns in Table 1. Once the reduced form number is known, one can consult the reference table of the 44 reduced forms [7] to obtain the appropriate transformation matrix to determine the conventional cell. In the last column, the frequency of occurrence (for the first 2.4 years that *Acta Crystallographica Section E* has been in existence) of each reduced form is given. The frequencies reveal that reduced form numbers 39, 27, 10, 37, and 14 are the most common for the centered monoclinic lattices.

The crystal symmetry can never exceed the metric symmetry, but it can be less. However, by analyzing the crystallographic databases, it has been observed that the metric and crystal symmetry are almost always the same [20, 21]. This coincidence of crystal and metric symmetry continues to hold true in recently published structures. For example, a detailed analysis of the NRFs, for 205 centered monoclinic cells published in *Acta Crystallographica Section E*, revealed that in every case the crystal and metric symmetry are identical. This fact provides a basis for a reliable method for evaluation of the symmetry of crystalline compounds [20, 21]. For example, cases in which metric symmetry exceeds the crystal symmetry represent either misidentified symmetry [22] or something unusual in the crystal structure [23]. Furthermore, from inspection of the NRF, one may ascertain extra relationships (not required by one of the 44 reduced forms) among the dot products. The experimentalist (or user of cell data in the crystallographic databases) should be aware that any extra specialization in the NRF may signify an important fact: for example, that one has inadvertently determined a derivative cell of a lattice of higher symmetry. Finally, as an integral part of routine practice, it is suggested that the normalized reduced form be determined and checked against a table of reduced forms [7] to ascertain the highest possible metric symmetry, to check for extra specialization, and to determine the transformation matrix to a conventional cell.

### 2.2 Lattice Similarity Determination via Normalized Reduced Cells

The reduced cell has long played a practical role in lattice-matching strategies [24, 25, and 26]. Likewise, the normalized reduced cell can play a useful role in lattice-matching techniques. Lattice-matching methods based on the reduced cell are used to locate lattices or derivative lattices that have the same metric parameters. Lattice-matching techniques based on the normalized reduced cell are designed to find lattices that have *similar* metric parameters. The two strategies are conceptually analogous. To understand how the normalized reduced-cell strategy works, first we summarize the reduced cell strategy, which is in common use.

#### 2.2.1 Lattice-Matching Procedure Based on Reduced Cells

The basic identification strategy is to check the lattice of the unknown (or an existing lattice) against all lattices in a database for a match and then to exclude unwanted matches on the basis of chemical information. In this scheme, for example, an unknown crystal is selected and mounted on a single-crystal diffractometer and a unit cell is determined and reduced. The reduced cell is then checked against the file of known materials. If desired, one calculates derivative lattices, which are also reduced and checked against the file of known lattices.

Experience has shown that identification based on matching reduced cells is very straightforward and reliable. In fact research with the crystallographic databases has shown that the reduced cell coupled with the element types uniquely defines a material. Currently this identification strategy is used in association with several crystallographic databases that are distributed to the scientific community. It has also been integrated into automated single-crystal x-ray diffractometers [27]. Similarly, a registration-identification procedure based on reduced cells is used in the addition of new compounds to the Cambridge Crystallographic Database [28]. Further details on lattice matching, on a computer program for lattice matching, and on the calculation of derivative lattices have been published as an NBS Technical Note [25] and in Acta Crystallographica [26].

#### 2.2.2 Lattice-Matching Technique Based on Normalized Reduced Cells

**Fig. 1:**
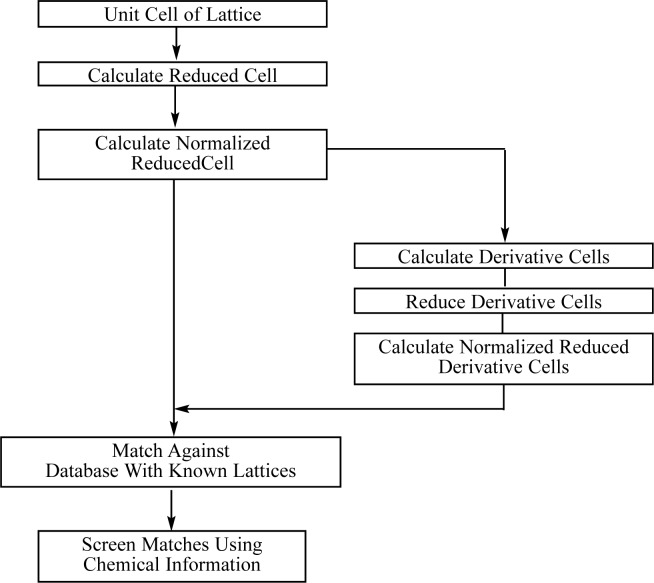
Determination of Similar Lattices via Lattice Matching (LM). Version of Lattice Matching based on matching the normalized reduced cell of an *unknown* against a *database* of known materials represented by their respective normalized reduced cells.

In a manner strictly parallel to the above, the normalized reduced cell can be used instead of the reduced cell in lattice-matching techniques. This is illustrated in Fig. 1 in which the normalized reduced cell has replaced the reduced cell. Here the basic search strategy is the same as above except that the normalized reduced cell is checked against the file (database) of normalized reduced cells for known materials. If desired, one calculates derivative lattices, which are also reduced, normalized and checked against the file of known lattices represented by their respective normalized reduced cells. The set of matches can be further restricted using chemical or other types of data. As most materials crystallize in the low symmetry crystal systems (e.g., over 90 % of organic and organometallic compounds crystallize in the triclinic, monoclinic, and orthorhombic systems), this type of lattice matching generally produces a limited and meaningful set of matches.

With this technique, the experimentalist can find metrically similar lattices. Knowledge of similar lattices has practical value in solving structures, in relating structures, in assignment of structure types, in materials design, and in nanotechnology. For example, information gained from a similarity search is valuable in the development of materials having a desired physical property. If a given compound has the desired property, one can find all compounds with similar lattices, some of which may exhibit the specified property to a greater extent.

## 3. Conclusion

The normalized reduced form and cell represent practical mathematical tools for the analysis of intraand interlattice relationships. With respect to intralattice relationships, experience with thousands of lattices has revealed that the normalized reduced form is a very useful tool for the evaluation of lattice-metric symmetry as well as for the determination of other significant relationships between the elements of the reduced form. Likewise, with respect to interlattice relationships, experience has shown that the normalized reduced cell is an excellent tool to determine metrically similar lattices.

In deducing significant interlattice relationships, one can systematically run the normalized reduced cell and reduced cell search in parallel with each other. It is suggested that such dual searching be routinely carried out—in data evaluation, in searching crystallographic databases, and in determining crystal structures—to ascertain the manner in which an extant or new lattice is related to the field of existing lattices. First, to find lattices that are metrically the same, the reduced cell can be matched against a file of reduced cells of known materials, and second, to find lattices that are metrically similar, the normalized reduced cell can be checked against a file of normalized reduced cells.

## Figures and Tables

**Table 1:** Metric classification of the 13 reduced forms that correspond to the centered monoclinic lattices. For each of the 13 generic reduced form matrices, a typical example of a normalized reduced form is given in columns 5–7. The cell data used in the calculations as well as the frequency data given in column 11 are based on data published in recent issues of *Acta Crystallographica, Sect. E*

Reduced form No.	Reduced form matrix			Normalized reduced form matrix			Type	Bravais lattice	Ref.	Freq.
*a* = *b*										
10	***a·a***	***a·a***	***c·c***	1.00	1.00	1.40	+	MC^a^	[8]	28
	***b·c***	***b·c***	***a·b***	0.24	0.24	0.46				
14	***a·a***	***a·a***	***c·c***	1.00	1.00	1.10	−	MC	[9]	21
	−|***b·c***|	−|***b·c***|	−|***a·b***|	−0.22	−0.22	−0.18				
17	***a·a***	***a·a***	***c·c***	1.00	1.00	5.65	−	MC	[10]	14
	−|***b·c***|	−|***a·c***|	−(***a·a***−|***b·c***|−|***a·c***|)	−0.29	−0.41	−0.30				
*b* = *c*										
20	***a·a***	***b·b***	***b·b***	1.00	1.27	1.27	+	MC	[11]	13
	***b·c***	***a·c***	***a·c***	0.26	0.19	0.19				
25	***a·a***	***b·b***	***b·b***	1.00	1.59	1.59	−	MI	[12]	12
	−|***b·c***|	−|***a·c***|	−|***a·c***|	−0.30	−0.43	−0.43				
*a* ≤ *b* ≤ *c*										
27	***a·a***	***b·b***	***c·c***	1.00	1.32	3.61	+	MC	[13]	32
	***b·c***	***a·a***/2	***a·a***/2	0.47	0.50	0.50				
28	***a·a***	***b·b***	***c·c***	1.00	1.31	1.95	+	MC	[14]	4
	***a·b***/2	***a·a***/2	***a·b***	0.16	0.50	0.32				
29	***a·a***	***b·b***	***c·c***	1.00	3.16	4.39	+	MC	[15]	5
	***a·c***/2	***a·c***	***a·a***/2	0.20	0.40	0.50				
30	***a·a***	***b·b***	***c·c***	1.00	2.20	2.75^b^	+	MC	^N/A^	0
	***b·b***/2	***a·b***/2	***a·b***	1.10	0.15	0.30				
37	***a·a***	***b·b***	***c·c***	1.00	1.10	1.55	−	MI	[16]	22
	−|***b·c***|	−***a·a***/2	0	−0.52	−0.50	0.00				
39	***a·a***	***b·b***	***c·c***	1.00	1.26	1.71	−	MC	[17]	47
	−|***b·c***|	0	−***a·a***/2	−0.30	0.00	−0.50				
41	***a·a***	***b·b***	***c·c***	1.00	1.24	9.36	−	MI	[18]	5
	−***b·b***/2	−|***a·c***|	0	−0.62	−0.36	0.00				
43	***a·a***	***b·b***	***c·c***	1.00	2.48	5.47	−	MI	[19]	2
	$-\frac{b\cdot b-\left|a\cdot b\right|}{2}$	$-\frac{a\cdot a-\left|a\cdot b\right|}{2}$	−|***a·b***|	−1.14	−0.40	−0.20				

a For each example, the first symbol “M” stands for monoclinic, and the second symbol “C or I” represents the centering of the conventional cell of the lattice.

b Created for illustrative purposes; an actual example was not found in *Acta Crystallogr., Sect. E.*

## Footnotes

1 Lattices that are defined by reduced cells that have corresponding angles equal and corresponding edges proportional.

2 A reduced form is said to be specialized if there is a simple mathematical relationship between two or more of the matrix elements (e.g. ***a·a*** = ***b·b***; ***b·c*** = *1/2* ***b·b***; ***b·c*** = *1/2* ***a·c***). Table 1 gives the types of specialization found in the reduced forms corresponding to the centered monoclinic lattices.
